# Forgotten but Striking: Complete Tricuspid Valve Degeneration Unmasked by Lancisi’s Sign

**DOI:** 10.7759/cureus.111876

**Published:** 2026-07-01

**Authors:** Gonzalo J Martinez-Ruiz, Nickolle A Cruz-Figueroa, Christopher Lopez, Abel E Rivera-Roman, Jorge J Ballester-Maldonado

**Affiliations:** 1 Internal Medicine, Universidad Central del Caribe, Bayamón, PRI; 2 Cardiology, Pavia Hospital, Arecibo, PRI

**Keywords:** awareness of cardiovascular disease, echocardiography, heart failure, lancisi’s sign, pulmonary edema, tricuspid regurgitation, valvular heart disease

## Abstract

Tricuspid regurgitation (TR) is increasingly recognized as an important cause of right-sided heart failure and adverse clinical outcomes. Although severe TR typically presents with a holosystolic murmur, advanced disease may produce atypical findings that obscure the diagnosis. Lancisi’s sign, characterized by prominent systolic jugular venous pulsations, remains an important but underrecognized bedside clue to severe TR. We present the case of a 60-year-old man with a remote history of presumed infective endocarditis who presented with progressive dyspnea, anasarca, and peripheral edema. Physical examination revealed marked systolic jugular venous pulsations consistent with Lancisi’s sign but no audible cardiac murmur. Transthoracic echocardiography demonstrated complete degeneration of the tricuspid valve leaflets, torrential TR, massive right-sided chamber dilation, and right atrial ventricularization. Doppler interrogation confirmed rapid pressure equalization between the right atrium and right ventricle. Given the advanced right-sided remodeling and evidence of end-organ involvement, the patient was deemed unsuitable for surgical or transcatheter intervention and was managed medically. This case highlights the diagnostic value of careful physical examination and illustrates how severe TR may present without its classic auscultatory findings. Early recognition of bedside signs such as Lancisi’s sign may facilitate timely diagnosis before progression to irreversible right-sided heart failure.

## Introduction

Tricuspid regurgitation (TR) refers to retrograde blood flow from the right ventricle into the right atrium due to incomplete valve closure. Once considered benign, it is now recognized as a neglected but clinically important disorder, affecting over 1.6 million patients in the United States and serving as an independent predictor of right-sided heart failure, recurrent hospitalizations, and mortality [1,2]. Most cases of TR are functional (secondary), driven by right ventricular dilation or pulmonary hypertension, which stretches the annulus and tethers the leaflets. Primary TR, which is less common, results from intrinsic valve pathology such as infective endocarditis, rheumatic involvement, trauma, or damage from intracardiac devices [3].

Clinically, TR often progresses silently until patients develop right-sided heart failure. On physical examination, TR typically produces a holosystolic murmur at the left lower sternal border that intensifies with inspiration. Another characteristic physical finding is Lancisi’s sign, which refers to prominent systolic jugular venous pulsations produced by giant c-v waves in patients with severe TR [4]. Although infrequently emphasized in contemporary clinical practice, it remains an important bedside finding that may facilitate recognition of advanced TR, particularly when auscultatory findings are subtle or absent.

Echocardiography remains the gold standard for diagnosing TR, providing a detailed assessment of valve anatomy, regurgitation severity, right ventricular function, and pulmonary pressures [5]. Management ranges from diuretics for symptom control to surgical or transcatheter intervention in advanced cases [6,7]. Prognosis is determined largely by the timing of recognition and the degree of right ventricular dysfunction at the time of intervention [1,2].

## Case presentation

A 60-year-old man with a history of hypertension, coronary artery disease, congestive heart failure, chronic liver disease, chronic obstructive pulmonary disease, chronic kidney disease stage 3a, remote intravenous drug use, and a reported history of infective endocarditis in the early 2000s presented with progressive shortness of breath, worsening lower-extremity edema, and generalized anasarca. Details regarding the original episode of infective endocarditis, including the causative organism, antimicrobial therapy, valve involvement, and prior echocardiographic findings, were unavailable. He had experienced recurrent hospitalizations in recent years for fatigue, volume overload, and pulmonary congestion. Aside from these symptoms, he denied chest pain, palpitations, dizziness, syncope, or other acute complaints.

**Figure 1:**
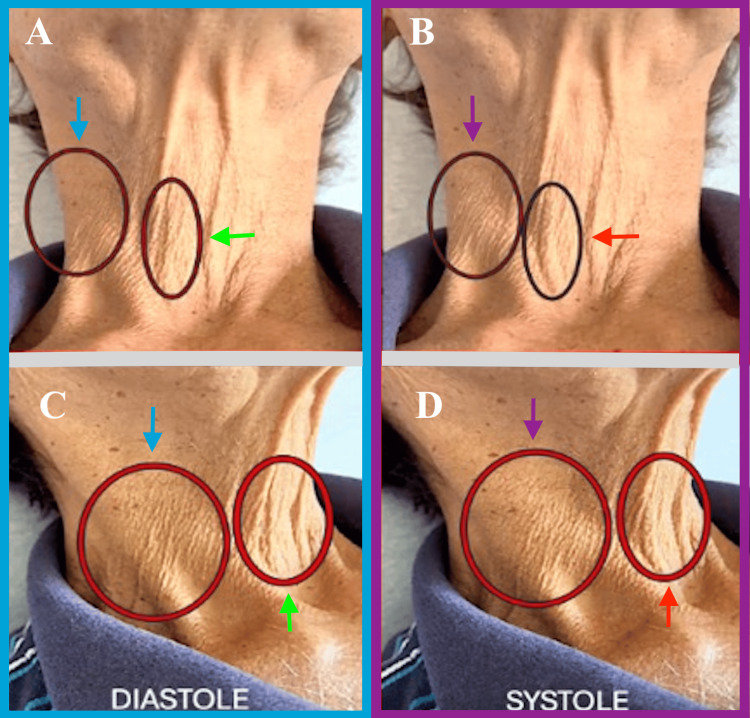
Dynamic jugular venous distension during systole and diastole in severe TR Panel A shows an anterior view of the neck during diastole. The larger circle (blue arrow) identifies the external jugular vein, and the smaller oval (green arrow) identifies the anterior jugular vein, both demonstrating minimal venous distension. Panel B shows the same anterior view during systole. The larger circle (purple arrow) identifies the external jugular vein, and the smaller oval (red arrow) identifies the anterior jugular vein. Marked systolic distension of both veins is evident. Panel C shows a lateral view of the neck during diastole. The larger circle (blue arrow) identifies the external jugular vein, and the smaller circle (green arrow) identifies the anterior jugular vein, with only mild venous distension. Panel D shows the lateral view during systole. The larger circle (purple arrow) identifies the external jugular vein, and the smaller circle (red arrow) identifies the anterior jugular vein. Marked systolic distension of both veins is evident, consistent with giant c-v waves (Lancisi’s sign) secondary to severe TR. TR, tricuspid regurgitation

**Figure 2:**
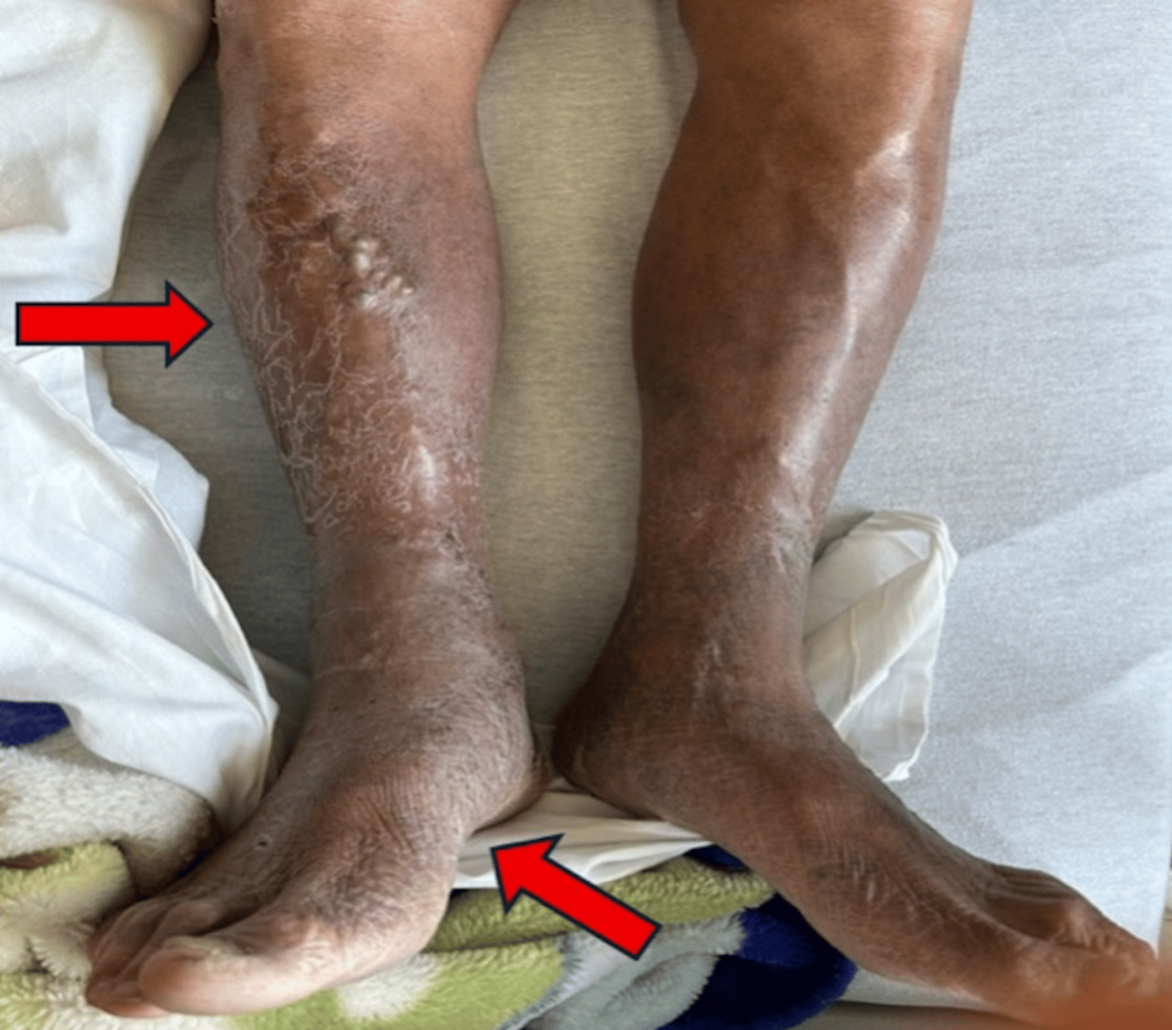
Lower-extremity manifestation of chronic right-sided heart failure Clinical photograph demonstrating bilateral lower-extremity edema, hyperpigmentation, and stasis dermatitis with scaling (red arrows), consistent with long-standing systemic venous hypertension secondary to severe TR and chronic right-sided heart failure. TR, tricuspid regurgitation

On physical examination, the patient appeared chronically ill, markedly cachectic, and frail, with evidence of severe chronic wasting; however, he remained hemodynamically stable (blood pressure: 133/77 mmHg, heart rate: 88 beats/minute, respiratory rate: 19 breaths/minute, and temperature: 36.1°C). Examination of the neck revealed markedly elevated jugular venous pulsations with prominent systolic venous waves visible at rest and extending to the angle of the mandible while the patient was in a semi-recumbent position (Figure 1, Video 1). Cardiac examination demonstrated a markedly laterally displaced and diffuse point of maximal impulse extending beyond the midclavicular line. Cardiac auscultation revealed a regular rhythm without an appreciable murmur. Pulmonary examination demonstrated bibasilar crackles. Additional findings included hepatomegaly, bilateral pitting edema, and chronic venous stasis changes of the lower extremities (Figure 2).

**Video 1:** Neck examination demonstrating marked jugular venous pulsations Clinical examination of the neck demonstrating marked systolic venous pulsations. Pronounced distension of the external and anterior jugular veins is visible during systole, with pulsations extending from the clavicular region to the angle of the mandible. These prominent systolic venous pulsations correspond to giant c-v waves produced by severe TR. The amplitude of the venous pulsations increases during systole and decreases during diastole, reflecting transmission of systolic pressure into the systemic venous circulation. TR, tricuspid regurgitation

Investigations

**Figure 3:**
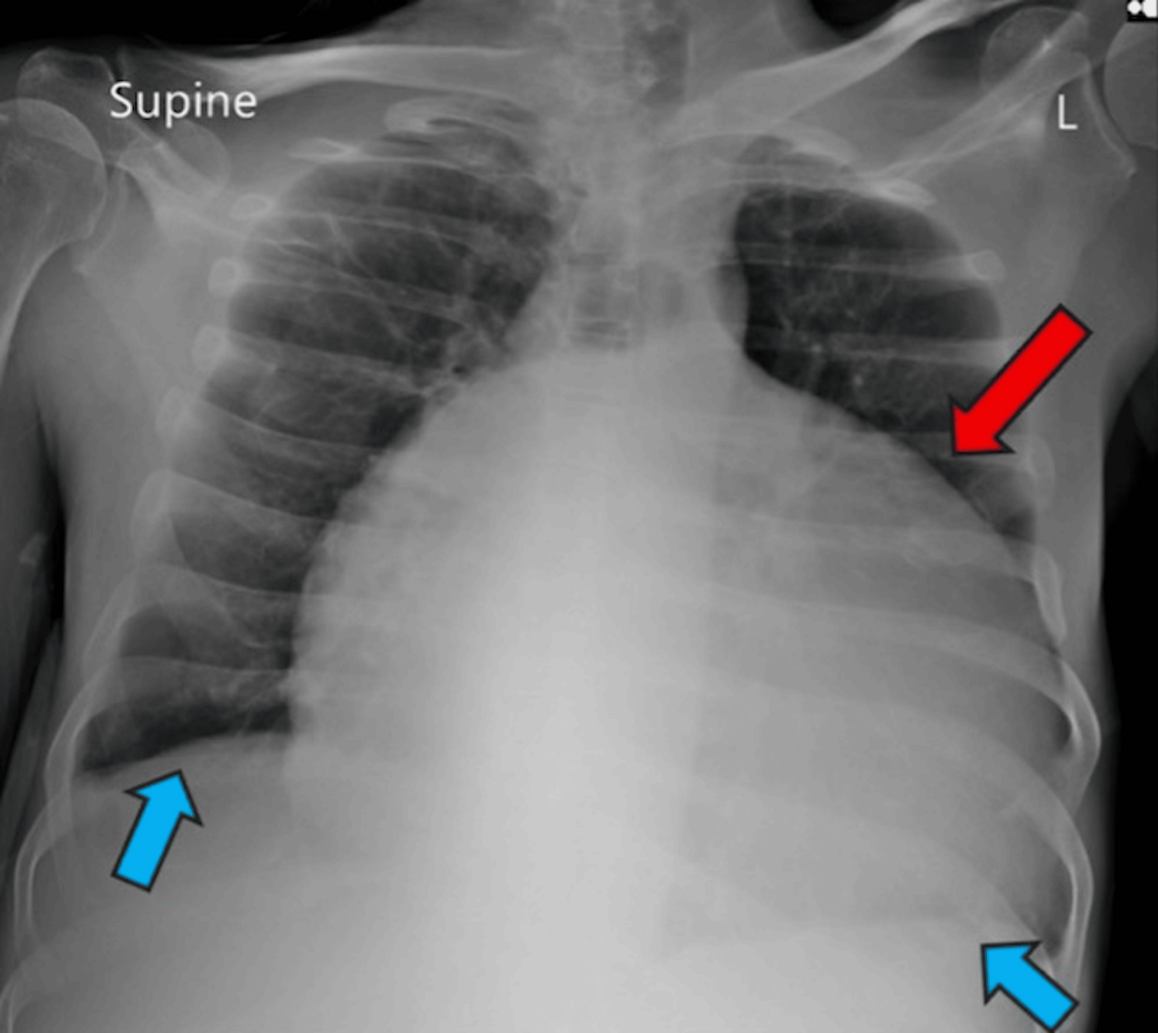
Chest radiograph showing cardiomegaly and pulmonary vascular congestion Portable anteroposterior chest radiograph obtained in the supine position, showing marked enlargement of the cardiac silhouette (red arrow), bilateral pulmonary vascular/interstitial congestion, and flattening of the diaphragms consistent with chronic hyperinflation (blue arrows). No focal air-space consolidation or pleural effusion is identified.

**Figure 4:**
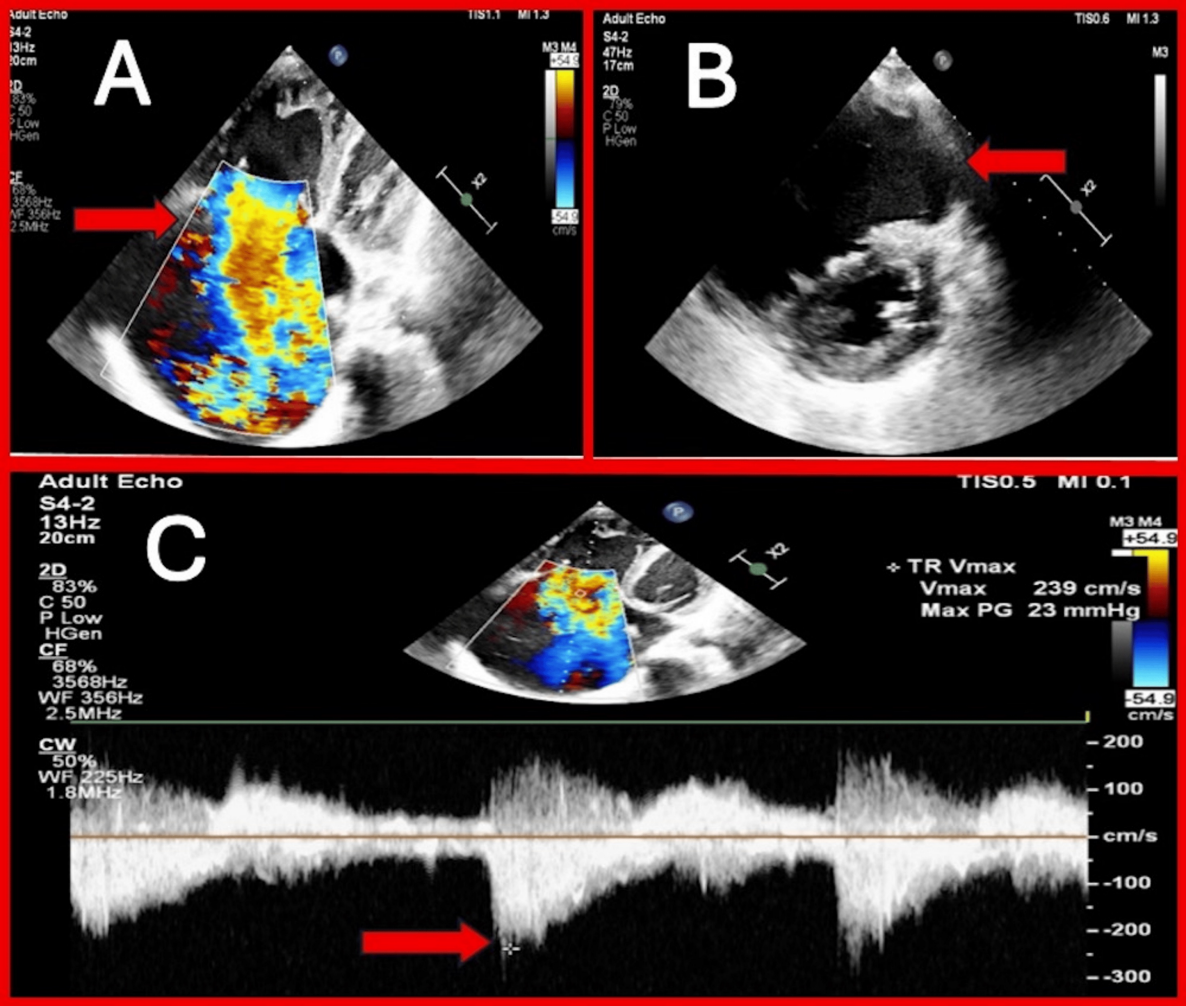
Echocardiographic findings in end-stage primary TR Panel A shows an apical four-chamber transthoracic echocardiogram with color Doppler demonstrating torrential TR. The red arrow highlights a broad systolic regurgitant jet extending from the right ventricle into the massively dilated right atrium. Panel B shows a parasternal short-axis view demonstrating severe right ventricular enlargement (red arrow). Panel C shows continuous-wave Doppler interrogation demonstrating a dense triangular regurgitant envelope with a peak velocity of 2.39 m/s (red arrow), consistent with severe TR and rapid right ventricular-right atrial pressure equalization during systole. TR, tricuspid regurgitation

Chest radiography demonstrated marked cardiomegaly with bilateral pulmonary vascular congestion (Figure 3). Transthoracic echocardiography (TTE) was subsequently performed for further evaluation (Video 2). The TTE revealed the absence of the tricuspid valve apparatus with torrential TR, accompanied by massive right atrial enlargement and severe right ventricular dilation (Figure 4A, 4B). Right ventricular systolic function was reduced, as evidenced by a tissue Doppler systolic velocity of <9.5 cm/s. Continuous-wave Doppler demonstrated a dense holosystolic regurgitant signal with a peak velocity of 2.39 m/s, consistent with rapid right ventricular-right atrial pressure equalization in the setting of torrential TR (Figure 4C). Pulmonary acceleration time was shortened to 82 ms, suggestive of pulmonary hypertension (Table 1) [8,9].

**Video 2:** TTE demonstrating TR TTE demonstrating complete absence of the tricuspid valve apparatus with unrestricted systolic flow between the right ventricle and right atrium, resulting in torrential TR. Marked right-sided chamber enlargement and loss of normal valvular coaptation are evident. TR, tricuspid regurgitation; TTE, transthoracic echocardiography

**Table 1:** Echocardiographic findings and clinical interpretation TTE demonstrated complete absence of the tricuspid valve apparatus with torrential TR, resulting in massive RA (9.85 cm) and severe RV (5.94 cm) enlargement, reflecting longstanding right-sided volume overload. TAPSE was not reported because severe TR can falsely exaggerate annular excursion and overestimate true RV systolic function. Despite a seemingly normal TR peak velocity (2.39 m/s) and low calculated gradient (22.85 mmHg), these measurements are misleading in the setting of free TR, where rapid RV-RA pressure equalization blunts the Doppler signal and underestimates the true systolic pressure gradient [8]. Conversely, the shortened RVOT acceleration time (82 ms) is suggestive of elevated pulmonary artery pressures and may provide a more reliable hemodynamic indicator in this setting [9]. LVOT, left ventricular outflow tract; RA, right atrium/right atrial; RV, right ventricle/right ventricular; RVOT, right ventricular outflow tract; TAPSE, tricuspid annular plane systolic excursion; TR, tricuspid regurgitation; TTE, transthoracic echocardiography; Vmax, maximum velocity

Parameter	Result	Reference range	Units	Interpretation
RA diameter	9.85 (High)	Normal: <5.4; Severe: >6.3	cm	Severe RA enlargement
RV mid-cavity diameter	5.94 (High)	Normal: 1.9-3.5; Severe: >4.2	cm	Severe RV dilation
Tricuspid valve morphology	Absent valve apparatus	Normal leaflet coaptation	-	Complete tricuspid valve destruction
TR severity	Torrential	None/trace	-	Severe primary TR
TAPSE	-	≥17	mm	Not reported; unreliable in torrential TR
TR peak velocity (Vmax)	2.39	Normal: <2.8; Severe: >3.6	m/s	Dense holosystolic regurgitant signal
TR peak gradient	22.85	-	mmHg	Rapid RV-RA pressure equalization
RVOT acceleration time	82 (Low)	Normal: >105; Severe: <60	ms	Suggestive of pulmonary hypertension
Left atrial dimension	4	2.7-4.0	cm	Upper limit of normal
Aortic root diameter	3	2.0-3.7	cm	Normal
LVOT diameter	2	1.8-2.4	cm	Normal

Management and hospital course

The patient was treated with aggressive intravenous diuresis, sodium and fluid restriction, and optimization of guideline-directed medical therapy. During hospitalization, he achieved a sustained negative fluid balance of approximately 1.5 L/day, with progressive improvement in dyspnea, peripheral edema, and overall volume status. Laboratory parameters demonstrated a favorable response to treatment, including improvement in B-type natriuretic peptide, serum sodium, potassium, chloride, and serum osmolality, reflecting successful decongestion and correction of volume overload-related abnormalities (Table 2). Persistent mild hyperbilirubinemia and transaminitis were attributed to chronic hepatic congestion and underlying cirrhosis. Given the extent of right-sided chamber remodeling, systemic venous congestion, and evidence of end-organ involvement, no surgical or transcatheter intervention was pursued. He was discharged on optimized medical therapy with close outpatient follow-up.

**Table 2:** Laboratory findings at admission and discharge Admission studies demonstrated findings consistent with significant volume overload and systemic venous congestion, including markedly elevated BNP, mild hyponatremia, hyperkalemia, elevated bilirubin, and elevated liver enzyme levels. Following aggressive diuretic therapy, substantial improvement was observed in BNP levels, serum sodium, potassium, chloride, and serum osmolality, reflecting successful decongestion. Persistent mild hyperbilirubinemia and transaminitis were consistent with underlying cirrhosis. Serum creatinine remained within the normal range; however, the results should be interpreted with caution because the patient was markedly cachectic, and reduced muscle mass may have underestimated the degree of renal dysfunction. Elevated serum bicarbonate (CO₂) was likely multifactorial and may reflect chronic carbon dioxide retention associated with underlying chronic obstructive pulmonary disease. Overall, the laboratory trends paralleled the patient’s clinical improvement following treatment of severe right-sided heart failure. Day 1 corresponds to admission laboratory values, and Day 7 corresponds to discharge laboratory values. ALP, alkaline phosphatase; ALT, alanine aminotransferase; AST, aspartate aminotransferase; BNP, B-type natriuretic peptide; BUN, blood urea nitrogen; Cl, chloride; CO₂, bicarbonate (total carbon dioxide); Cr, creatinine; GFR, glomerular filtration rate; HCT, hematocrit; Hgb, hemoglobin; K, potassium; LDH, lactate dehydrogenase; Na, sodium; Osm, serum osmolality; PLT, platelet count

Lab test	Result: Day 1	Result: Day 7	Reference range	Units	Interpretation
WBC	4.9	7.8	4.8-11.0	× 10³/µL	Within normal limits
Hgb	11.5	13.1	11.0-15.1	g/dL	Within normal limits
HCT	36.6	38.4	34.5-43.8	%	Within normal limits
PLT	139 (Low)	143 (Low)	161-400	× 10³/µL	Persistent mild thrombocytopenia
Glucose	129 (High)	108 (High)	74-106	mg/dL	Persistent mild hyperglycemia
BUN	11	13	7.0-18	mg/dL	Within normal limits
Cr	0.73	0.49 (Low)	0.53-1.02	mg/dL	Stable serum creatinine
GFR	60	>60	>60	mL/min/1.73 m²	Stable renal function
Osm	265 (Low)	281.3	275.0-295.0	mOsm/kg H₂O	Hypo-osmolality on admission, resolved
Na⁺	132 (Low)	139	136-145	mmol/L	Mild hyponatremia, resolved
K⁺	6.1 (High)	4.4	3.5-5.1	mmol/L	Moderate hyperkalemia, resolved
Cl⁻	96 (Low)	109	98-107	mmol/L	Mild hypochloremia, resolved
CO₂	32.9 (High)	21.9	21.0-32.0	mmol/L	Elevated bicarbonate on admission, resolved
BNP	876.3 (High)	413.0 (High)	0-100.0	pg/mL	Markedly elevated BNP, improved with treatment
Total bilirubin	1.54 (High)	1.62 (High)	0.20-1.00	mg/dL	Persistent hyperbilirubinemia
AST	147 (High)	141 (High)	15-37	U/L	Persistent AST elevation
ALT	43	45	16-61	U/L	Within normal limits
ALP	136	145	87-241	U/L	Within normal limits
LDH	968 (High)	699 (High)	0-200	U/L	Markedly elevated, with mild improvement

Follow-up

Following discharge, the patient reported improvement in symptoms and functional status. Although he required two hospitalizations during the subsequent six months for recurrent volume overload, the frequency and severity of symptoms were reduced compared with the period preceding diagnosis, when hospital admissions occurred nearly monthly. He continues to receive outpatient management focused on symptom control and prevention of recurrent decompensation.

## Discussion

This case represents an uncommon end-stage manifestation of primary TR characterized by complete destruction of the tricuspid valve apparatus, resulting in torrential regurgitation, massive right-sided chamber remodeling, and functional “ventricularization” of the right atrium. In the absence of functional tricuspid leaflets, the normal systolic pressure gradient between the right ventricle and right atrium was effectively abolished, allowing unrestricted transmission of right ventricular systolic pressure into the right atrium and systemic venous circulation. Consequently, the right atrium functioned as a direct extension of the right ventricle during systole, producing the striking clinical finding of Lancisi’s sign.

**Figure 5:**
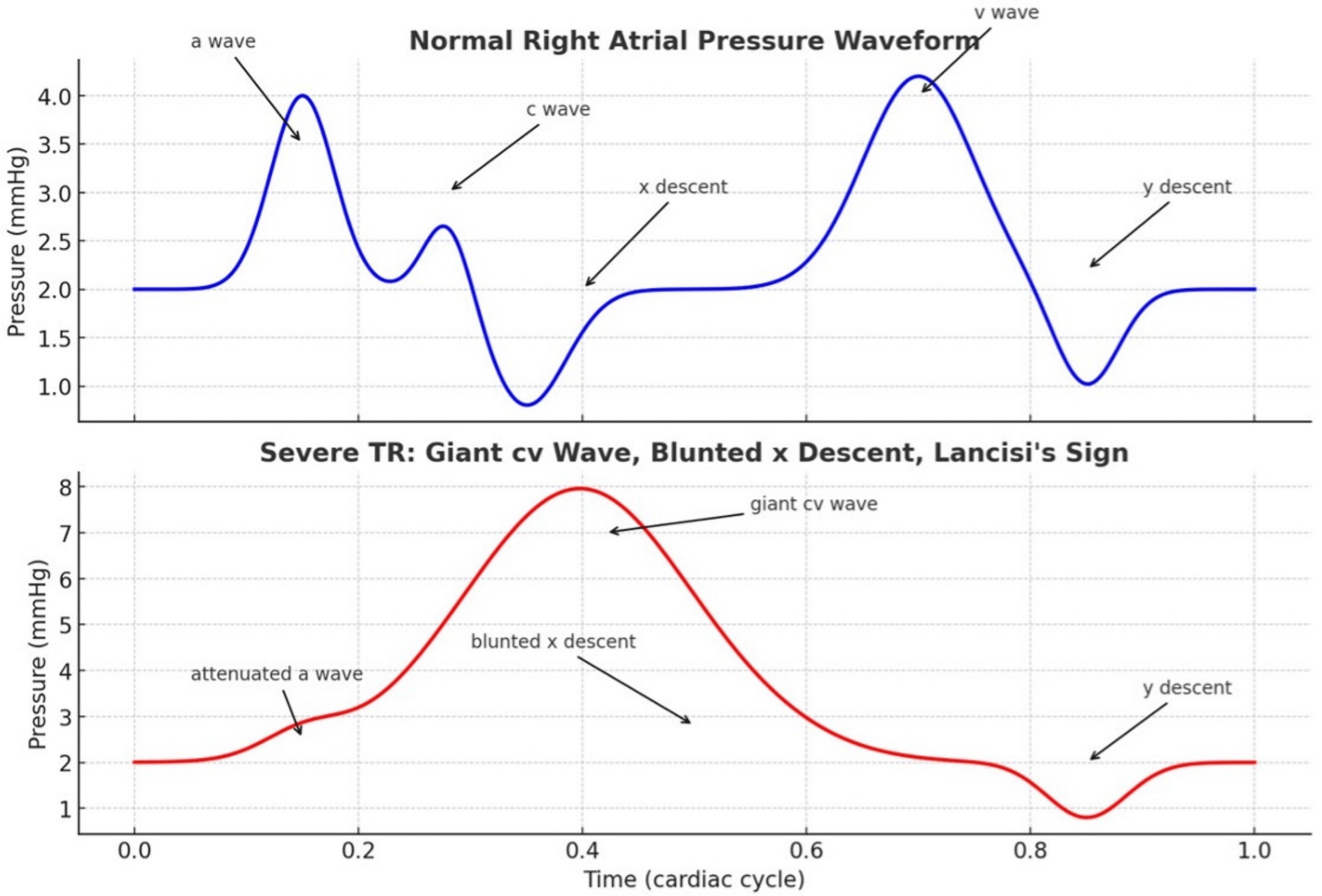
Hemodynamic basis of Lancisi’s sign: right atrial pressure waveform in severe TR Right atrial pressure waveform in severe TR. Comparison of a normal right atrial pressure waveform (top) and severe TR (bottom). Severe TR is characterized by a giant systolic c-v wave resulting from regurgitant flow into the right atrium, with blunting or loss of the x descent. The prominent systolic venous pulsations produced by the giant c-v wave correspond clinically to Lancisi’s sign. TR, tricuspid regurgitation

The hemodynamic basis of this phenomenon is reflected in the jugular venous pulse waveform. Under normal conditions, the jugular venous pulse consists of distinct a, c, and v waves with corresponding x and y descents. In severe TR, regurgitant flow into the right atrium during systole results in fusion of the c and v waves into a prominent systolic c-v wave, accompanied by attenuation or loss of the x descent (Figure 5) [4]. These hemodynamic abnormalities were clinically evident in our patient as marked systolic jugular venous pulsations (Video 1).

Lancisi’s sign in the absence of a murmur

One of the most unusual aspects of this case was the absence of a characteristic cardiac murmur despite torrential TR. Classically, TR presents with a holosystolic murmur at the left lower sternal border that increases with inspiration (Carvallo’s sign) [10]. However, murmur intensity does not necessarily correlate with disease severity. In advanced TR, progressive equalization of right ventricular and right atrial pressures may reduce the velocity of regurgitant flow and diminish the generation of audible turbulence. Furthermore, complete destruction of the tricuspid valve apparatus likely altered normal intracardiac flow dynamics, further limiting murmur production. This uncommon presentation highlights the limitations of relying solely on auscultatory findings and underscores the enduring value of careful bedside examination. In our patient, prominent systolic jugular venous pulsations provided a more reliable clue to the severity of the underlying valvular disease than cardiac auscultation.

Clinical implications and timing of intervention

Once regarded as a relatively benign valvular lesion, severe TR is now recognized as an important contributor to heart failure, recurrent hospitalization, end-organ dysfunction, and mortality [1-3]. Progressive volume overload leads to right-sided heart failure, systemic venous hypertension, hepatic congestion, renal dysfunction, atrial arrhythmias, and reduced survival [1,2]. Although most contemporary cases of severe TR are functional in origin, the patient’s reported history of remote infective endocarditis suggested this as the presumed inciting event, resulting in progressive degeneration and eventual complete loss of the tricuspid valve apparatus [3]. Because prior microbiologic and echocardiographic records were unavailable, this etiology could not be definitively confirmed. Reports describing such extensive valve destruction are limited in the published literature, making this case an uncommon illustration of the potential long-term consequences of presumed healed infective endocarditis.

Management of severe TR remains challenging, and outcomes are highly dependent on the timing of intervention. Current American College of Cardiology (ACC)/American Heart Association (AHA) and European Society of Cardiology/European Association for Cardio-Thoracic Surgery guidelines support surgical intervention in appropriately selected patients with severe primary TR before the development of advanced right ventricular dysfunction, severe pulmonary hypertension, or irreversible end-organ damage [6,7]. According to the 2020 ACC/AHA guidelines, tricuspid valve surgery carries a Class I recommendation for patients with severe TR undergoing left-sided valve surgery. In patients with isolated severe primary TR who remain symptomatic despite optimal medical therapy, isolated tricuspid valve surgery carries a Class IIa recommendation because it may improve symptoms and reduce recurrent hospitalizations. Surgery may also be considered in asymptomatic patients with severe primary TR and progressive right ventricular dilation or dysfunction, corresponding to a Class IIb recommendation [6]. Unfortunately, our patient presented with massive right atrial and right ventricular dilation, chronic systemic venous congestion, and evidence of end-organ involvement, findings that placed him beyond the optimal therapeutic window for intervention. Consequently, both surgical and transcatheter therapies were considered unlikely to provide meaningful clinical benefit, and management was limited to aggressive diuresis and optimization of guideline-directed medical therapy.

## Conclusions

This case illustrates a catastrophic end stage of TR, in which complete valve destruction resulted in right atrial ventricularization and impressive hemodynamic findings despite the absence of an audible murmur. The silent presentation highlights the limitations of relying solely on auscultation and underscores the enduring value of careful bedside examination, where Lancisi’s sign proved more revealing than the stethoscope.

Unfortunately, by the time of presentation, the opportunity for intervention had already passed, leaving medical therapy as the only feasible treatment strategy. Severe TR, long regarded as a benign entity, carries a poor prognosis when recognition and referral are delayed. The tricuspid valve can no longer remain the “forgotten valve”; early recognition and timely intervention may determine the difference between preserved life and inevitable decline.
